# Relationship between systemic inflammatory response index and bone mineral density in children and adolescents aged 8-19 years: a cross-sectional study based on NHANES 2011-2016

**DOI:** 10.3389/fendo.2025.1537574

**Published:** 2025-02-25

**Authors:** Dejun Cun, Nan Yang, Lin Zhou, Wenxing Zeng, Bin Chen, Zichen Pan, Huang Feng, Ziwei Jiang

**Affiliations:** ^1^ Department of Traditional Chinese Medicine, The First Clinical College of Guangzhou University of Chinese Medicine, Guangzhou, China; ^2^ Department of Traditional Chinese Medicine, The First Clinical College of Yunnan University of Traditional Chinese Medicine, Kunming, China; ^3^ College of First Clinical Medicine, Shandong University of Traditional Chinese Medicine, Jinan, Shandong, China; ^4^ Department of Lower Limb Trauma Orthopedics, The First Affiliated Hospital of Guangzhou University of Traditional Chinese Medicine, Guangzhou, China

**Keywords:** systemic inflammatory response index, bone mineral density, children and adolescents, NHANES, cross-sectional study

## Abstract

**Objective:**

This study aims to investigate the relationship between the Systemic Inflammatory Response Index (SIRI) and bone mineral density (BMD) in children and adolescents aged 8-19 years.

**Methods:**

A cross-sectional design was used, utilizing NHANES data from 2011-2016, including 3,205 participants aged 8 to 19 years. Weighted multivariable regression analysis was conducted to assess the association between SIRI and BMD at the lumbar spine, pelvis, trunk, and whole body. Additionally, smooth curve fitting was applied to examine the nonlinear relationship between SIRI and BMD, and subgroup analyses were performed to explore potential interaction effects and modifiers.

**Results:**

SIRI was significantly positively correlated with BMD at the pelvis, trunk, and whole body (p < 0.05). After adjusting for covariates, a one-unit increase in ln(SIRI) was associated with increases in BMD of 0.018 g/cm², 0.006 g/cm², and 0.005 g/cm² for the pelvis, trunk, and whole body, respectively. Nonlinear analysis revealed a saturation effect between ln(SIRI) and BMD, with a more pronounced impact at specific threshold values. Subgroup analysis indicated that gender, age, BMI and total calcium levels modulated the relationship between SIRI and BMD.

**Conclusion:**

SIRI is significantly associated with BMD in children and adolescents, with a positive effect on BMD at specific threshold levels. This finding suggests that SIRI may serve as a potential biomarker for assessing the risk of low bone mineral density, offering theoretical support for the prevention and intervention of bone health issues such as osteoporosis.

## Introduction

BMD is a crucial indicator of bone health and fracture risk (1), particularly during childhood and adolescence, which are critical periods for peak bone mass (PBM) development (2). PBM represents the highest bone density in an individual’s lifetime and has been shown to be a key determinant of osteoporosis and fragility fractures in later life (3, 4). Childhood and adolescence are key stages for maximizing bone accumulation, with optimal bone growth during puberty not only determining adult bone health but also providing some protection against age-related bone loss and osteoporosis-related diseases (5, 6). However, the factors influencing bone health are multifaceted (7), including genetic, nutritional, hormonal, and environmental influences.

In recent years, systemic inflammation has increasingly been recognized as an important factor influencing bone remodeling (8, 9). Systemic inflammation may promote bone resorption by activating osteoclasts while inhibiting osteoblast differentiation and function, leading to reduced bone formation. This mechanism is particularly pronounced in chronic inflammatory states and may have profound effects on bone homeostasis and structural integrity. The Systemic Inflammatory Response Index (SIRI), introduced by Qi et al. in 2016 (10), is a novel inflammatory marker calculated based on neutrophil, lymphocyte, and monocyte counts. SIRI can distinguish between the immune-inflammatory responses of three different pathways in the body, reflecting the overall state of inflammation and immune balance. Initially used to predict the survival of pancreatic cancer patients undergoing gemcitabine chemotherapy, SIRI has since been expanded to assess mortality, severity, and sepsis risk in stroke patients (11), and as a reliable immune-inflammatory marker to differentiate between myelin oligodendrocyte glycoprotein antibody-associated diseases (MOGAD) and aquaporin-4 immunoglobulin G-positive neuromyelitis optica spectrum disorders (NMOSD) (12). Furthermore, studies have shown that SIRI is significantly associated with various chronic diseases, such as cardiovascular diseases, metabolic disorders, and chronic inflammation (13, 14). However, the potential impact of SIRI on bone health during the rapid growth phases of children and adolescents remains underexplored. Adolescence is a critical period for rapid skeletal growth, with significant bone turnover, and inflammation may uniquely and importantly affect this process. Yet, research on the relationship between SIRI and BMD in children and adolescents is still lacking.

The National Health and Nutrition Examination Survey (NHANES) provides nationally representative data to study the association between SIRI and BMD. This study utilizes NHANES data from 2011 to 2016 to explore the relationship between SIRI levels and BMD in U.S. children and adolescents aged 8-19 years, with an in-depth analysis of nonlinear relationships, subgroup differences, saturation effects, and threshold effects. The findings will elucidate the potential mechanisms by which SIRI influences skeletal development, providing scientific evidence to optimize bone health and reduce future fracture risk. We hypothesize that, within a moderate range of inflammation levels, elevated SIRI is associated with increased BMD in children and adolescents.

## Methods

### Study design and population

This study is based on data from the 2011-2016 National Health and Nutrition Examination Survey (NHANES), utilizing a cross-sectional design. NHANES is an ongoing survey program led by the National Center for Health Statistics (NCHS), aimed at obtaining nationally representative samples of the health and nutrition status of the U.S. civilian, non-institutionalized population through complex, multi-stage probability sampling. The survey includes various demographic subgroups, encompassing different ages, genders, races/ethnicities, and socioeconomic backgrounds, ensuring the data’s broad representativeness and validity. NHANES data are collected through both household interviews and mobile examination centers (MECs). During the household interview phase, respondents answer a range of questions regarding health, socioeconomic status, lifestyle, and other factors to provide detailed background information. Subsequently, participants visit the MEC for comprehensive physical exams and laboratory tests, including blood analysis and dual-energy X-ray absorptiometry (DXA), among others. This rigorous process enables NHANES to accurately assess health indicators and provide consistent biomarker data. Comprehensive NHANES information can be accessed at http://www.cdc.gov/nhanes.

**Figure 1:**
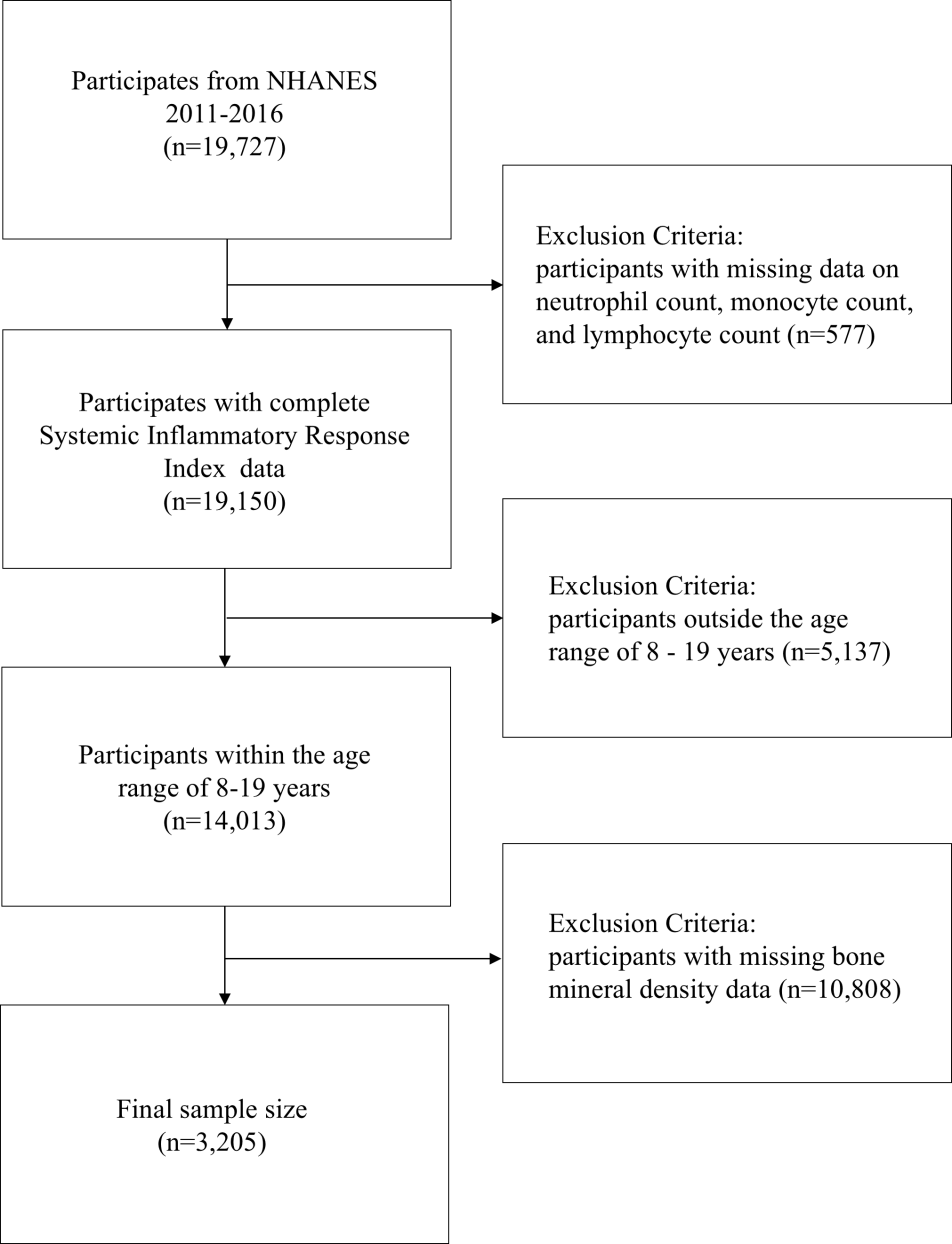
Participant inclusion and exclusion criteria flowchart for NHANES.

Data for this study were obtained from three cycles of NHANES (2011-2016), involving 19,727 participants. After excluding 577 participants due to the lack of systemic inflammatory response index (SIRI) data, 5,137 participants who were outside the age range of 8-19 years, and 10,590 participants without available BMD data, the final study sample consisted of 3,205 participants (**Figure 1**). This study adhered to the Strengthening the Reporting of Observational Studies in Epidemiology (STROBE) guidelines for reporting (15).

### Definition of variables

#### Systemic inflammatory response index

**Figure 2:**
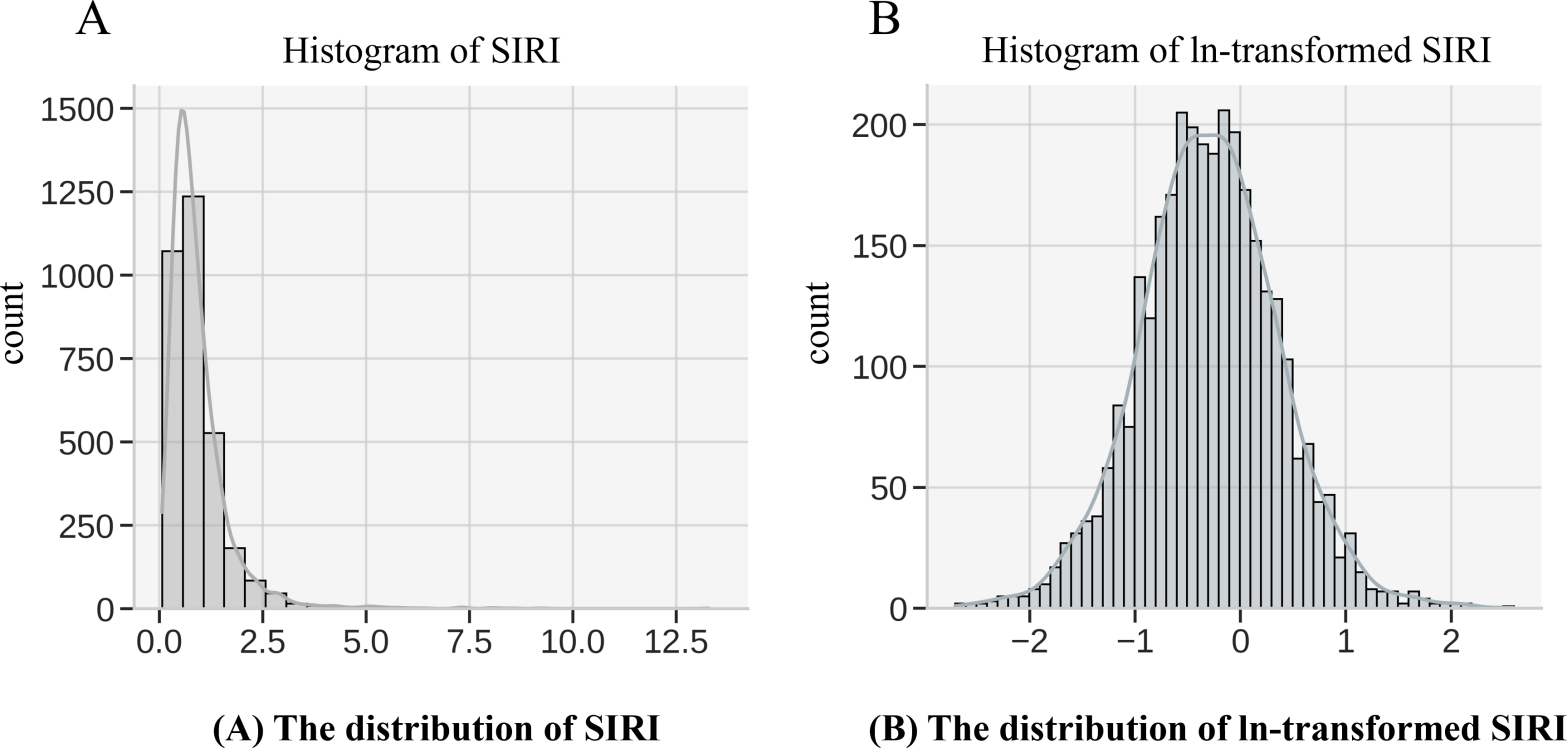
Logarithmic transformation of SIRI. **(A)** SIRI **(B)** ln(SIRI).

The Systemic Inflammatory Response Index (SIRI) is the primary exposure variable in this study, calculated based on the counts of neutrophils, monocytes, and lymphocytes. The formula for calculating SIRI in this study is as follows: (Neutrophil count × Monocyte count)/Lymphocyte count. These white blood cell count data were derived from the complete blood count (CBC) and white blood cell differentiation tests in NHANES. NHANES laboratory tests adhere to strict standards for sample collection, processing, and analysis. Blood samples were obtained via venipuncture and processed in laboratories within the mobile examination centers (MECs). CBC and white blood cell differentiation tests were conducted using automated blood analyzers that are regularly calibrated and subject to the quality control standards of the National Health and Nutrition Examination Survey (NHANES) to ensure data accuracy and consistency. Given that inflammatory markers typically exhibit a skewed distribution, a natural logarithmic transformation (ln) of SIRI was applied (**Figure 2**) to normalize the data, reduce the influence of extreme values, and meet the assumptions for statistical analysis. The transformed ln(SIRI) was then used in multivariable regression models to assess its association with BMD.

### Lumbar, pelvis, trunk, and total bone mineral density

The outcome variable in this study is BMD, which includes measurements at four specific sites: lumbar spine (Lumbar BMD), pelvis (Pelvis BMD), trunk (Trunk BMD), and total body (Total BMD). BMD was assessed using dual-energy X-ray absorptiometry (DXA) with a Hologic Discovery model densitometer (Hologic, Inc., Bedford, Massachusetts) and analyzed using Apex version 3.2 software. All BMD values were expressed in grams per square centimeter (g/cm²) and were standardized according to NHANES procedures. BMD data were collected and processed by trained technicians, and the standardized data can be accessed in the NHANES dataset.

### Covariates

The selection of covariates was based on theoretical reasoning and research on the relationship between inflammation and bone health. Covariates included age, sex, race/ethnicity, the ratio of family income to poverty (PIR), the number of days per week with at least 60 minutes of physical activity, serum 25-hydroxyvitamin D (X25.OH.D), phosphorus, total calcium, and alkaline phosphatase (ALP). Physical activity specifically refers to activities that elevate the participant’s heart rate and result in slight breathlessness. With the exception of sex, race/ethnicity, and the number of days of at least 60 minutes of physical activity per week, all other covariates were treated as continuous variables. Race/ethnicity was categorized into five groups: non-Hispanic White, non-Hispanic Black, Mexican American, other Hispanic, and other ethnicities. The number of days per week with at least 60 minutes of physical activity was categorized from 0 to 7 days. Detailed measurement techniques for the study variables can be found on the Centers for Disease Control and Prevention (CDC) website at www.cdc.gov/nchs/nhanes/.

### Statistical analysis

All statistical analyses were conducted using R software version 4.2.1 and EmpowerStats 2.0. To minimize bias introduced by missing data, the random forest imputation method was used to fill in missing values. Descriptive statistics were used to analyze the basic characteristics of the sample, with continuous variables expressed as mean ± standard deviation or median (interquartile range), and categorical variables presented as frequencies and percentages. To compare baseline characteristics, weighted linear regression was applied to continuous variables to ensure sample representativeness and improve the accuracy of estimates, while weighted chi-square tests were used for categorical variables to better reflect the distribution of the target population. In multivariable regression analysis, the SIRI was log-transformed (ln) to correct for its skewed distribution. The transformed ln(SIRI) was treated as the primary exposure variable and grouped into quartiles: Quartile 1 (Q1) for values below the 25th percentile, Quartile 2 (Q2) for the 25th to 50th percentile, Quartile 3 (Q3) for the 50th to 75th percentile, and Quartile 4 (Q4) for the 75th to 100th percentile. The association between ln(SIRI) in each quartile and BMD at the lumbar spine, pelvis, trunk, and total body was assessed, and regression coefficients (β values) along with 95% confidence intervals (CI) were calculated. To control for potential confounders, three progressively adjusted regression models were constructed: Model 1 (unadjusted), Model 2 (adjusted for age, sex, and race/ethnicity), and Model 3 (further adjusted for PIR, the number of days per week of at least 60 minutes of physical activity, X25.OH.D, phosphorus, total calcium, and ALP). To explore potential effect modification by age, sex, race/ethnicity, total calcium, and ALP in the relationship between ln(SIRI) and BMD, total calcium and ALP were categorized into low, medium, and high levels for stratified analysis, and interaction tests were conducted in the models to assess potential modifying effects at different levels. Threshold effect analysis was performed to determine the saturation point in the relationship between ln(SIRI) and BMD. Finally, sensitivity analyses were performed to ensure the robustness of the results. All statistical tests were two-sided, with a p-value < 0.05 considered statistically significant.

### Ethics and consent

This study utilized publicly available data from the National Health and Nutrition Examination Survey (NHANES), conducted by the National Center for Health Statistics (NCHS), and approved by the NCHS Research Ethics Review Board. All NHANES participants provided written informed consent prior to data collection, and the study protocol adhered to the ethical principles outlined in the Declaration of Helsinki.

## Results

### Basic characteristics of participants

A total of 3,205 participants aged 8 to 19 years were included in this study, with a mean age of 13.11 ± 3.44 years. Of these, 52.45% were male and 47.55% were female. The average ln(SIRI) of all participants was -0.30 ± 0.66. The mean BMD values for the lumbar spine, pelvis, trunk, and total body were 0.87 ± 0.19 g/cm², 1.05 ± 0.23 g/cm², 0.77 ± 0.16 g/cm², and 0.95 ± 0.16 g/cm², respectively. Significant differences in baseline characteristics, including age, race, PIR, total calcium, phosphorus, ALP, and the number of days per week of at least 60 minutes of physical activity, were observed across the quartiles of ln(SIRI) (all p < 0.05). Compared to the lowest quartile (Q1), Participants in the highest quartile (Q4) were significantly older and had higher BMI indices, more likely to be non-Hispanic White (p < 0.001), and engaged in more days of at least 60 minutes of physical activity per week. Furthermore, participants in the Q4 group had lower levels of total calcium, phosphorus, ALP, and PIR compared to those in the Q1 group (**Table 1**).

**Table 1:** Baseline Characteristics of Participants Stratified by Quartiles of ln(SIRI).

Characteristic	Quartiles of ln (SIRI)				P-Value
	Q1	Q2	Q3	Q4	
	N=801	N = 799	N = 801	N = 804	
Age,(years)	12.51 ± 3.31	12.82 ± 3.41	13.20 ± 3.42	13.90 ± 3.47	<0.001
Sex, (%)					0.186
Male	55.06	53.32	51.56	49.88	
Female	44.94	46.68	48.44	50.12	
Race/ethnicity, (%)					<0.001
Mexican American	15.73	20.90	22.85	26.12	
Other Hispanic	7.24	12.02	13.48	16.42	
Non-Hispanic White	18.98	26.41	28.84	27.11	
Non-Hispanic Black	41.95	23.78	19.35	16.29	
Other Race	16.10	16.90	15.48	14.05	
PIR	2.13 ± 1.51	1.98 ± 1.46	1.99 ± 1.46	1.88 ± 1.40	0.009
Total calcium, (mg/dL)	9.61 ± 0.24	9.60 ± 0.22	9.60 ± 0.25	9.54 ± 0.26	<0.001
Phosphorus, (mg/dL)	4.72 ± 0.61	4.64 ± 0.59	4.55 ± 0.59	4.37 ± 0.63	<0.001
ALP, (IU/L)	208.18 ± 98.78	192.53 ± 97.85	177.65 ± 102.05	154.55 ± 90.45	<0.001
25(OH)D, (nmol/L)	57.29 ± 19.37	58.68 ± 19.41	59.41 ± 20.57	57.66 ± 20.06	0.130
BMI, (kg/m^2^)	20.65 ± 4.92	21.90 ± 5.51	23.00 ± 5.99	24.05 ± 6.75	<0.001
Days physically active at least 60 min, (%)					<0.001
0	3.12	3.00	2.37	3.73	
1	1.87	2.63	2.75	2.86	
2	2.87	3.25	4.12	3.23	
3	9.86	10.89	15.11	16.17	
4	24.47	24.53	20.85	24.63	
5	19.35	22.15	18.48	19.78	
6	3.12	4.26	6.99	4.48	
7	35.33	29.29	29.34	25.12	
Lumbar BMD, (g/cm^2^)	0.84 ± 0.19	0.85 ± 0.19	0.87 ± 0.20	0.90 ± 0.19	<0.001
Pelvis BMD, (g/cm^2^)	1.01 ± 0.23	1.04 ± 0.24	1.07 ± 0.23	1.10 ± 0.23	<0.001
Trunk BMD, (g/cm^2^)	0.75 ± 0.15	0.76 ± 0.16	0.78 ± 0.16	0.80 ± 0.15	<0.001
Total BMD, (g/cm^2^)	0.93 ± 0.16	0.93 ± 0.16	0.95 ± 0.16	0.97 ± 0.15	<0.001

### Analysis of the relationship between BMD and SIRI

**Table 2** presents the results of the weighted multivariable regression analysis, showing a significant positive association between SIRI levels and BMD in all models, except for lumbar BMD (p < 0.05). After adjusting for all covariates, each unit increase in ln(SIRI) was associated with a significant increase in pelvic, trunk, and total body BMD. Specifically, pelvic BMD increased by 0.018 g/cm², trunk BMD increased by 0.006 g/cm², and total body BMD increased by 0.005 g/cm².

**Table 2:** Association between ln(SIRI) and Bone Mineral Density.

Exposure	Model 1[β (95%CI)]	Model 2[β (95%CI)]	Model 3[β (95%CI)]
Lumbar BMD (continuous)	0.038 (0.028, 0.048)	0.011 (0.004, 0.017)	0.004 (-0.002, 0.010)
Lumbar BMD (quartile)			
Quartile 1	Reference	Reference	Reference
Quartile 2	0.020 (0.002, 0.039)	0.015 (0.003, 0.027)	0.011 (-0.001, 0.022)
Quartile 3	0.054 (0.036, 0.073)	0.025 (0.013, 0.036)	0.016 (0.004, 0.027)
Quartile 4	0.080 (0.061, 0.098)	0.028 (0.016, 0.040)	0.016 (0.005, 0.028)
p for trend	<0.00001	0.00123	0.20331
Pelvis BMD (continuous)	0.056 (0.044, 0.069)	0.025 (0.016, 0.033)	0.018 (0.010, 0.027)
Pelvis BMD (quartile)			
Quartile 1	Reference	Reference	Reference
Quartile 2	0.041 (0.018, 0.064)	0.035 (0.019, 0.050)	0.029 (0.013, 0.044)
Quartile 3	0.073 (0.050, 0.095)	0.039 (0.024, 0.055)	0.030 (0.014, 0.045)
Quartile 4	0.109 (0.086, 0.132)	0.050 (0.034, 0.065)	0.037 (0.021, 0.052)
p for trend	<0.00001	<0.00001	0.00002
Trunk BMD (continuous)	0.032 (0.024, 0.040)	0.011 (0.005, 0.016)	0.006 (0.001, 0.012)
Trunk BMD (quartile)			
Quartile 1	Reference	Reference	Reference
Quartile 2	0.020 (0.005, 0.036)	0.016 (0.006, 0.026)	0.012 (0.003, 0.022)
Quartile 3	0.042 (0.027, 0.057)	0.020 (0.010, 0.030)	0.014 (0.004, 0.023)
Quartile 4	0.063 (0.048, 0.079)	0.023 (0.013, 0.033)	0.015 (0.005, 0.025)
p for trend	<0.00001	0.00014	0.02017
Total BMD (continuous)	0.031 (0.023, 0.039)	0.010 (0.005, 0.015)	0.005 (0.000, 0.010)
Total BMD (quartile)			
Quartile 1	Reference	Reference	Reference
Quartile 2	0.020 (0.005, 0.035)	0.017 (0.007, 0.026)	0.013 (0.004, 0.022)
Quartile 3	0.040 (0.025, 0.055)	0.020 (0.011, 0.029)	0.014 (0.005, 0.023)
Quartile 4	0.061 (0.046, 0.076)	0.021 (0.012, 0.031)	0.013 (0.004, 0.022)
p for trend	<0.00001	0.00010	0.03120

Model 1: Unadjusted. Model 2: Adjusted for age, sex, and race. Model 3: Adjusted for age, sex, race, PIR, physical activity (≥60 minutes/week), X25.OH.D, phosphorus, total calcium, and ALP.

Further analysis was conducted by categorizing ln(SIRI) from a continuous variable into quartiles (Q1 to Q4). Compared to the lowest quartile (Q1), the highest quartile (Q4) was associated with increases in pelvic, trunk, and total body BMD of 0.037 g/cm² (β: 0.037; 95% CI: 0.021–0.052, p < 0.00001), 0.015 g/cm² (β: 0.015; 95% CI: 0.005–0.025, p = 0.00326), and 0.013 g/cm² (β: 0.013; 95% CI: 0.004–0.022, p = 0.00559), respectively. These findings further support a positive association between SIRI and BMD. However, after separately adjusting for BMI and body weight, the effect of SIRI on BMD was no longer significant (P > 0.05). This suggests that BMI and body weight may play a more significant role in predicting BMD, potentially masking the independent effect of SIRI (**Supplementary Tables 1**, **2**).

### Subgroup analysis and interaction tests

The study results indicate that the association between SIRI levels and pelvic, trunk, and total BMD varied across subgroups (**Tables 3**). In the subgroup analysis stratified by sex, the correlation between ln(SIRI) and pelvic and total BMD showed significant differences (p for interaction < 0.05). Specifically, for each one-unit increase in ln(SIRI), pelvic BMD increased by 0.0271 g/cm² (β: 0.0271; 95% CI: 0.0160–0.0382, p < 0.0001) and total BMD increased by 0.0100 g/cm² (β: 0.0100; 95% CI: 0.0036–0.0164, p = 0.0023) in males. However, in females, the increase in ln(SIRI) did not have a significant effect on either pelvic or total BMD (p > 0.05). Trunk BMD, on the other hand, did not show any significant differences (p for interaction > 0.05). In the subgroup analysis stratified by BMI, the correlation between ln(SIRI) and total BMD showed significant differences (p for interaction < 0.05). In the BMI ≥ 25 kg/m² group, the relationship between SIRI and total BMD was significant (P < 0.05), with each one-unit increase in ln(SIRI) associated with a decrease of 0.013 g/cm² in total BMD (β: -0.013; 95% CI: -0.022, -0.003; p = 0.0087). Moreover, interaction tests revealed that race, age, total calcium, ALP, and the number of physical activity days per week did not significantly influence the association between SIRI and BMD in the stratified analyses (**Table 3**, all p for interaction > 0.05).

**Table 3:** Subgroup analysis of the association between ln(SIRI) and bone mineral density, adjusted for age, sex, race, PIR, physical activity (≥60 minutes/week), X25.OH.D, phosphorus, total calcium, BMI and ALP.

Subgroup	Pelvis BMD [β (95%CI)]	P for interaction	Trunk BMD [β (95%CI)]	P for interaction	Total BMD [β (95%CI)]	P for interaction
Sex		0.0068		0.0508		0.0077
Male	0.027 (0.016, 0.038)		0.010 (0.003, 0.017)		0.010 (0.0036, 0.0164)	
Female	0.004 (-0.009, 0.017)		-0.0005 (-0.008, 0.007)		-0.003 (-0.0105, 0.0041)	
Race/ethnicity		0.4471		0.6184		0.6274
Mexican American	0.029 (0.006, 0.051)		0.008 (-0.007, 0.022)		0.008 (-0.005, 0.021)	
Other Hispanic	0.034 (0.004, 0.064)		0.020 (0.002, 0.039)		0.017 (0.000, 0.034)	
Non-Hispanic White	0.011 (-0.001, 0.023)		0.004 (-0.003, 0.012)		0.003 (-0.003, 0.010)	
Non-Hispanic Black	0.019 (-0.001, 0.040)		0.005 (-0.008, 0.018)		0.002 (-0.009, 0.014)	
Other Race	0.028 (-0.002, 0.058)		0.005 (-0.014, 0.024)		0.005 (-0.012, 0.022)	
Age		0.1475		0.0807		0.1873
8–10 years old	0.004 (-0.017, 0.024)		-0.004 (-0.016, 0.009)		-0.003 (-0.015, 0.008)	
11–13 years old	0.019 (0.002, 0.036)		0.004 (-0.006, 0.015)		0.009 (-0.001, 0.019)	
14–16 years old	0.033 (0.017, 0.048)		0.016 (0.006, 0.026)		0.013 (0.004, 0.022)	
17–19 years old	0.023 (0.009, 0.037)		0.010 (0.001, 0.019)		0.008 (-0.001, 0.016)	
Total calcium		0.4051		0.3583		0.3216
7.30–9.499	0.024 (0.008, 0.040)		0.009 (-0.001, 0.019)		0.002 (-0.007, 0.012)	
9.50–9.68	0.014 (0.000, 0.027)		0.003 (-0.005, 0.012)		0.005 (-0.003, 0.013)	
9.681–11.3	0.026 (0.012, 0.040)		0.012 (0.003, 0.020)		0.011 (0.003, 0.019)	
ALP		0.2386		0.0963		0.5211
31-105	0.015 (0.002, 0.028)		0.002 (-0.006, 0.010)		0.003 (-0.004, 0.011)	
106-240.4	0.027 (0.014, 0.040)		0.013 (0.005, 0.022)		0.009 (0.001, 0.016)	
240.52-740	0.012 (-0.002, 0.026)		0.002 (-0.007, 0.011)		0.004 (-0.005, 0.012)	
BMI		0.1903		0.0300		0.0036
BMI< 18.5	-0.001(-0.015,0.012)		-0.003 (-0.013,0.006)		-0.003 (-0.012,0.005)	
BMI ≥ 18.5 & < 25	0.008 (-0.002,0.018)		0.006(-0.001,0.013)		0.006 (-0.000, 0.013)	
BMI ≥ 25	-0.008 (-0.023,0.007)		-0.009 (-0.019, 0.001)		-0.013 (-0.022, -0.003)	
Days physically active at least 60 min		0.2848		0.1641		0.1251
0	-0.034 (-0.096, 0.027)		-0.023 (-0.062, 0.015)		-0.018 (-0.055, 0.018)	
1	0.013 (-0.051, 0.077)		0.002 (-0.038, 0.042)		0.003 (-0.034, 0.041)	
2	0.065 (0.008, 0.122)		0.047 (0.011, 0.082)		0.036 (0.002, 0.069)	
3	0.018 (-0.010, 0.045)		-0.001 (-0.018, 0.016)		-0.002 (-0.018, 0.014)	
4	0.014 (-0.004, 0.031)		0.004 (-0.007, 0.015)		0.002 (-0.008, 0.012)	
5	0.031 (0.015, 0.048)		0.012 (0.002, 0.022)		0.010 (0.001, 0.020)	
6	0.018 (-0.015, 0.051)		-0.002 (-0.023, 0.019)		-0.012 (-0.031, 0.008)	
7	0.016 (0.002, 0.030)		0.008 (-0.000, 0.017)		0.009 (0.001, 0.017)	

In the interaction test between BMI and SIRI, we performed subgroup analyses stratified by sex and age. The results showed that the interaction between SIRI and BMI had a significant effect on total BMD (p for interaction < 0.05). In the male group with BMI ≥ 18.5 & < 25, the relationship between SIRI and total BMD was significant (P < 0.05), with each one-unit increase in ln (SIRI) associated with an increase of 0.01 g/cm² in total BMD (β: 0.01; 95% CI: 0.001, 0.019). In the male group with BMI ≥ 25, each one-unit increase in ln (SIRI) was associated with a decrease of 0.015 g/cm² in total BMD (β: -0.015; 95% CI: -0.028, -0.001; P < 0.05). In the female group with BMI ≥ 25, each one-unit increase in ln (SIRI) was associated with a decrease of 0.017 g/cm² in total BMD (β: -0.017; 95% CI: -0.029, -0.004; P < 0.05). In other BMI groups, the effect of SIRI on total BMD was not significant (P > 0.05). In the male and female groups with BMI ≥ 25, the effect of SIRI on total BMD was statistically significant (P < 0.05). In the subgroup analysis stratified by age, in the 8–10 years age group, when BMI < 18.5, each one-unit increase in ln (SIRI) was associated with a decrease of 0.010 g/cm² in total BMD (95% CI: -0.019, -0.001, P < 0.05). No significant effect of SIRI on other BMD sites (Pelvis BMD, Trunk BMD) was observed (P > 0.05). When BMI ≥ 18, the effect of SIRI on all BMD sites was not significant (P > 0.05). In the 11–13 years age group, no significant effect of SIRI on any BMD sites was observed (P > 0.05). In the 14–16 years age group, when BMI ≥ 18.5 & < 25, each one-unit increase in ln (SIRI) was associated with an increase of 0.018 g/cm² in total BMD (95% CI: 0.005, 0.032, P < 0.05). When BMI < 18.5 & ≥ 25, the effect of SIRI on all BMD sites was not significant (P > 0.05). In the 17–19 years age group, when BMI ≥ 25, each one-unit increase in ln (SIRI) was associated with a decrease of 0.017 g/cm² in total BMD (95% CI: -0.033, -0.002, P < 0.05). When BMI < 25, the effect of SIRI on all BMD sites was not significant (P > 0.05) (**Table 4**).

**Table 4:** Interaction between ln SIRI and BMI, and subgroup analysis stratified by sex and age.

Subgroup	Pelvis BMD [β (95%CI)]	P for interaction	Trunk BMD [β (95%CI)]	P for interaction	Total BMD [β (95%CI)]	P for interaction
Stratified by sex						
Male		0.0550		0.0416		0.0094
BMI< 18.5	0.004 (-0.013,0.02)		-0.001(-0.013,0.011)		0.000 (-0.011, 0.012)	
BMI ≥ 18.5 & < 25	0.012 (-0.002, 0.026)		0.008 (-0.002, 0.018)		0.010 (0.001, 0.019)	
BMI ≥ 25	-0.018(-0.039, 0.003)		-0.014 (-0.029, 0.001)		-0.015(-0.028, -0.001)	
Female		0.4665		0.2187		0.0849
BMI< 18.5	-0.015 (-0.035, 0.005)		-0.010 (-0.024, 0.003)		-0.012(-0.025, 0.000)	
BMI ≥ 18.5 & < 25	-0.000 (-0.015, 0.015)		0.001 (-0.009, 0.011)		-0.001(-0.010, 0.009)	
BMI ≥ 25	-0.010 (-0.030, 0.010)		-0.011 (-0.024, 0.002)		-0.017(-0.029, -0.004)	
Stratified by age						
8–10 years old		0.4984		0.8719		0.4691
BMI< 18.5	-0.004 (-0.016, 0.009)		-0.006 (-0.015, 0.002)		-0.010(-0.019, -0.001)	
BMI ≥ 18.5 & < 25	-0.015 (-0.033, 0.003)		-0.005 (-0.017, 0.006)		-0.003(-0.016, 0.010)	
BMI ≥ 25	-0.018 (-0.066, 0.030)		-0.013 (-0.044, 0.017)		0.007 (-0.027, 0.041)	
11–13 years old		0.9661		0.6322		0.3273
BMI< 18.5	0.006 (-0.013, 0.025)		0.003 (-0.010, 0.015)		0.004 (-0.008, 0.016)	
BMI ≥ 18.5 & < 25	0.002 (-0.018, 0.022)		-0.003 (-0.017, 0.010)		0.010 (-0.003, 0.023)	
BMI ≥ 25	0.003 (-0.035, 0.041)		-0.009 (-0.035, 0.016)		-0.010(-0.034, 0.014)	
14–16 years old		0.3124		0.1934		0.0641
BMI< 18.5	0.016 (-0.023, 0.055)		-0.001 (-0.013, 0.011)		0.004 (-0.021, 0.030)	
BMI ≥ 18.5 & < 25	0.025 (0.004, 0.045)		0.008 (-0.002, 0.018)		0.018 (0.005, 0.032)	
BMI ≥ 25	-0.004 (-0.036, 0.028)		-0.014 (-0.029, 0.001)		-0.010(-0.031, 0.011)	
17–19 years old		0.2029		0.0617		0.0072
BMI< 18.5	0.033 (-0.027, 0.093)		0.019 (-0.023, 0.060)		0.030 (-0.008, 0.069)	
BMI ≥ 18.5 & < 25	0.012 (-0.002, 0.026)		0.011 (-0.004, 0.025)		0.010 (-0.004, 0.023)	
BMI ≥ 25	0.011 (-0.010, 0.032)		-0.013 (-0.030, 0.003)		-0.017(-0.033, -0.002)	

### Analysis of nonlinear, threshold, and saturation effects in the relationship between SIRI and bone mineral density

**Figure 3:**
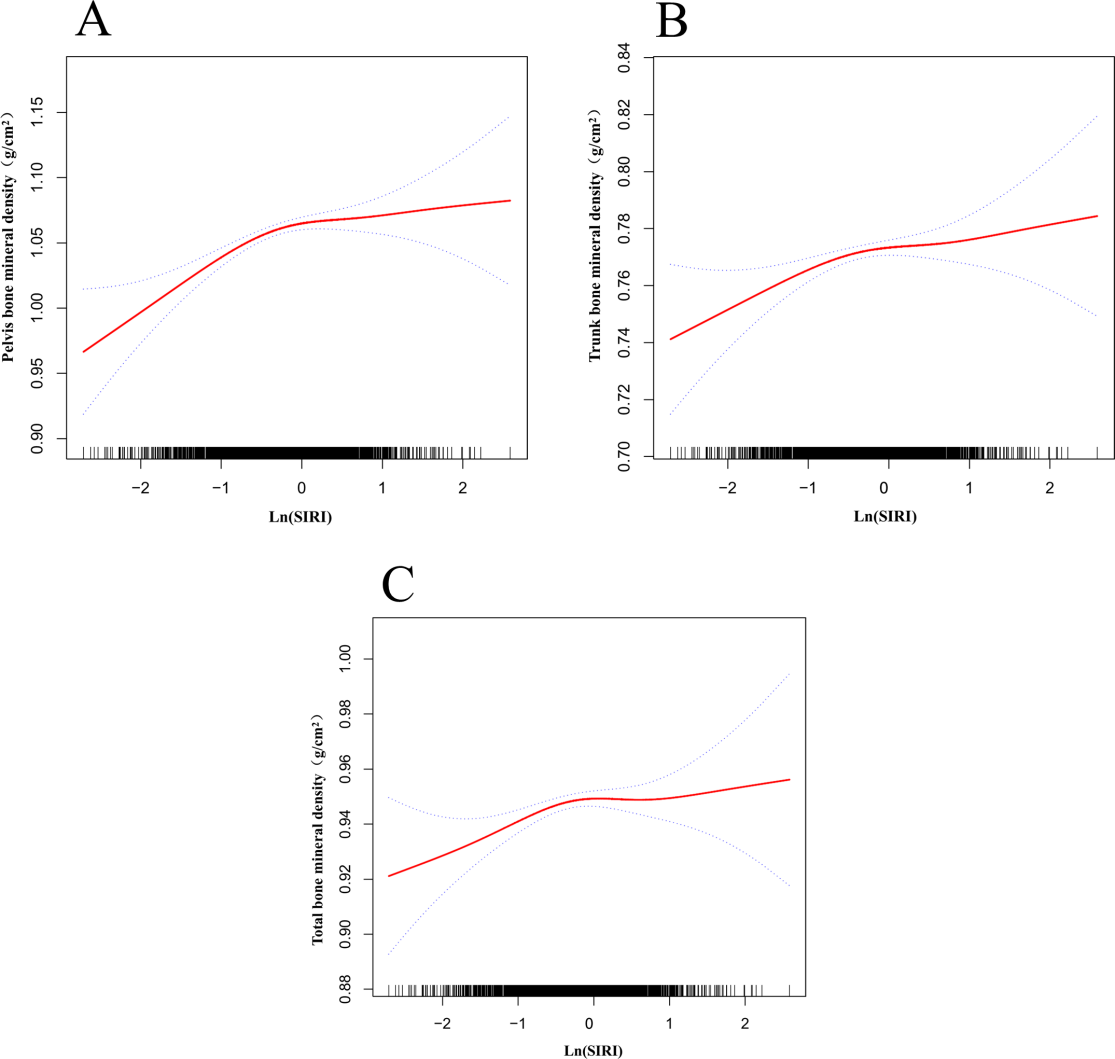
Nonlinear relationship between ln(SIRI) and bone mineral density. The red solid line represents the smooth curve fit, and the dashed lines indicate the 95% confidence interval. **(A)** Relationship between ln(SIRI) and pelvic BMD; **(B)** Relationship between ln(SIRI) and trunk BMD; **(C)** Relationship between ln(SIRI) and total BMD.

**Figure 3** shows the nonlinear relationship and saturation effects between ln (SIRI) and pelvic, trunk, and total BMD, as demonstrated by smooth curve fitting. Among all participants, the saturation point for the relationship between ln(SIRI) and pelvic BMD was -0.336 (**Table 5**). When ln(SIRI) was below -0.336, the effect size was 0.050 (95% CI: 0.032–0.067, p < 0.0001), indicating a significant positive effect of ln(SIRI) on pelvic BMD. However, when ln(SIRI) exceeded -0.336, the increase in ln(SIRI) no longer significantly affected pelvic BMD (p > 0.05), exhibiting a saturation effect. Similarly, for trunk BMD, the saturation point for ln(SIRI) was -0.258. When ln(SIRI) was below -0.258, the effect size was 0.018 (95% CI: 0.008–0.029, p = 0.0004), showing a significant positive correlation. However, when ln(SIRI) exceeded -0.258, the effect size became -0.004 (95% CI: -0.013, 0.005, p = 0.3749), indicating that the effect on trunk BMD was not significant. For total BMD, the saturation point for ln(SIRI) was -0.26. When ln(SIRI) was below -0.26, the effect size was 0.019 (95% CI: 0.010–0.028, p < 0.0001), significantly increasing total BMD. When ln(SIRI) exceeded -0.26, the effect size became -0.006 (95% CI: -0.015, 0.002, p = 0.1454), suggesting that the increase in ln(SIRI) no longer had a significant effect on total BMD. In summary, ln(SIRI) exhibited nonlinear and saturation effects on pelvic, trunk, and total BMD. with significant increases in BMD below the threshold and no significant effect above the threshold.

**Table 5:** Saturation and threshold effects of ln(SIRI) on bone mineral density, with stratified results by sex, age, and total calcium levels, adjusted for age, sex, race, PIR, days of at least 60 minutes of physical activity per week, X25.OH.D, phosphorus, total calcium, and ALP.

Subgroup	Pelvis BMD [β (95%CI)]	P for interaction	Trunk BMD [β (95%CI)]	P for interaction	Total BMD [β (95%CI)]	P for interaction
Sex		0.0068		0.0508		0.0077
Male	0.027 (0.016, 0.038)		0.010 (0.003, 0.017)		0.010 (0.0036, 0.0164)	
Female	0.004 (-0.009, 0.017)		-0.0005 (-0.008, 0.007)		-0.003 (-0.0105, 0.0041)	
Race/ethnicity		0.4471		0.6184		0.6274
Mexican American	0.029 (0.006, 0.051)		0.008 (-0.007, 0.022)		0.008 (-0.005, 0.021)	
Other Hispanic	0.034 (0.004, 0.064)		0.020 (0.002, 0.039)		0.017 (0.000, 0.034)	
Non-Hispanic White	0.011 (-0.001, 0.023)		0.004 (-0.003, 0.012)		0.003 (-0.003, 0.010)	
Non-Hispanic Black	0.019 (-0.001, 0.040)		0.005 (-0.008, 0.018)		0.002 (-0.009, 0.014)	
Other Race	0.028 (-0.002, 0.058)		0.005 (-0.014, 0.024)		0.005 (-0.012, 0.022)	
Age		0.1475		0.0807		0.1873
8–10 years old	0.004 (-0.017, 0.024)		-0.004 (-0.016, 0.009)		-0.003 (-0.015, 0.008)	
11–13 years old	0.019 (0.002, 0.036)		0.004 (-0.006, 0.015)		0.009 (-0.001, 0.019)	
14–16 years old	0.033 (0.017, 0.048)		0.016 (0.006, 0.026)		0.013 (0.004, 0.022)	
17–19 years old	0.023 (0.009, 0.037)		0.010 (0.001, 0.019)		0.008 (-0.001, 0.016)	
Total calcium		0.4051		0.3583		0.3216
7.30–9.499	0.024 (0.008, 0.040)		0.009 (-0.001, 0.019)		0.002 (-0.007, 0.012)	
9.50–9.68	0.014 (0.000, 0.027)		0.003 (-0.005, 0.012)		0.005 (-0.003, 0.013)	
9.681–11.3	0.026 (0.012, 0.040)		0.012 (0.003, 0.020)		0.011 (0.003, 0.019)	
ALP		0.2386		0.0963		0.5211
31-105	0.015 (0.002, 0.028)		0.002 (-0.006, 0.010)		0.003 (-0.004, 0.011)	
106-240.4	0.027 (0.014, 0.040)		0.013 (0.005, 0.022)		0.009 (0.001, 0.016)	
240.52-740	0.012 (-0.002, 0.026)		0.002 (-0.007, 0.011)		0.004 (-0.005, 0.012)	
Days physically active at least 60 min		0.2848		0.1641		0.1251
0	-0.034 (-0.096, 0.027)		-0.023 (-0.062, 0.015)		-0.018 (-0.055, 0.018)	
1	0.013 (-0.051, 0.077)		0.002 (-0.038, 0.042)		0.003 (-0.034, 0.041)	
2	0.065 (0.008, 0.122)		0.047 (0.011, 0.082)		0.036 (0.002, 0.069)	
3	0.018 (-0.010, 0.045)		-0.001 (-0.018, 0.016)		-0.002 (-0.018, 0.014)	
4	0.014 (-0.004, 0.031)		0.004 (-0.007, 0.015)		0.002 (-0.008, 0.012)	
5	0.031 (0.015, 0.048)		0.012 (0.002, 0.022)		0.010 (0.001, 0.020)	
6	0.018 (-0.015, 0.051)		-0.002 (-0.023, 0.019)		-0.012 (-0.031, 0.008)	
7	0.016 (0.002, 0.030)		0.008 (-0.000, 0.017)		0.009 (0.001, 0.017)	

**Figure 4:**
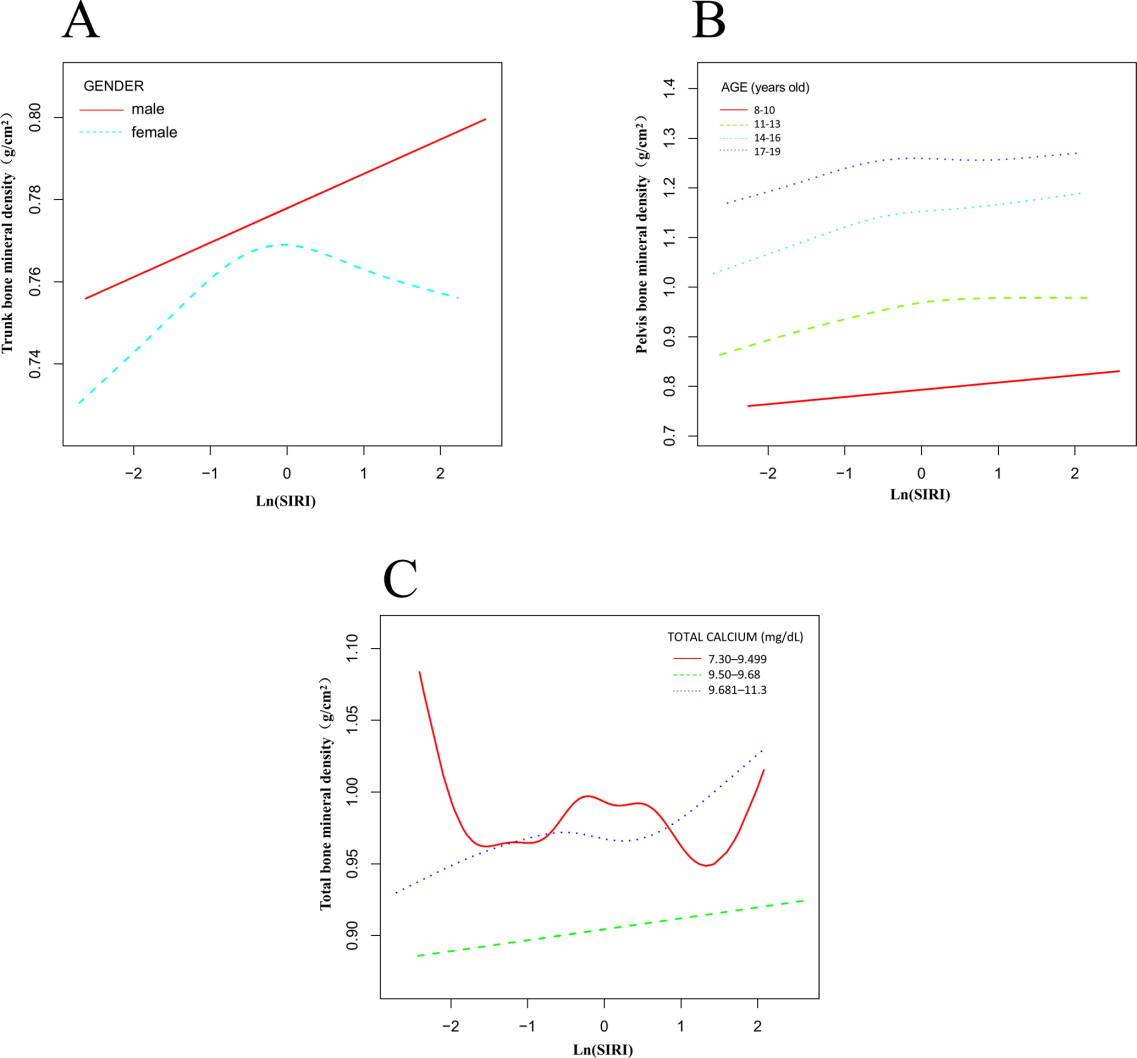
Nonlinear association between ln(SIRI) and bone mineral density stratified by sex, age, and total calcium levels. **(A)** Stratified by sex; **(B)** Stratified by age; **(C)** Stratified by total calcium levels.

All participants were grouped by sex, and smooth curve and threshold effect evaluations were conducted (**Table 5**, **Figure 4A**). The results showed that for trunk BMD in males, when ln(SIRI) < -0.357, each unit increase in ln(SIRI) was associated with an increase of 0.020 g/cm² in trunk BMD (95% CI: 0.005, 0.035, p = 0.0110). In contrast, for individuals with ln(SIRI) > -0.357, the correlation between ln(SIRI) and trunk BMD was not significant (β: 0.003; 95% CI: -0.010, 0.015, p = 0.6794). In females, the relationship between ln(SIRI) and trunk BMD displayed a significant threshold effect. When ln(SIRI) < -0.821, each unit increase in ln(SIRI) was associated with a significant increase in trunk BMD (β: 0.038; 95% CI: 0.012, 0.064, p = 0.0044). However, when ln(SIRI) exceeded -0.821, each unit increase in ln(SIRI) resulted in a decrease of 0.009 g/cm² in trunk BMD (β: -0.009; 95% CI: -0.018, -0.001, p = 0.0385), indicating that above this threshold, increases in ln(SIRI) may have a negative impact on female BMD. Participants were grouped by age into four categories (8-10 years, 11-13 years, 14-16 years, 17-19 years), and smooth curve and saturation effect evaluations were conducted (**Table 5**, **Figure 4B**). The analysis revealed that the saturation effect between ln(SIRI) and pelvic, trunk, and total BMD was most pronounced in the 14-16-year age group. For pelvic BMD, when ln(SIRI) < -0.233, each unit increase in ln(SIRI) was associated with an increase of 0.081 g/cm² (β: 0.081; 95% CI: 0.049, 0.114, p < 0.0001). In contrast, when ln(SIRI) > -0.233, the increase in ln(SIRI) did not significantly affect pelvic BMD (β: -0.016; 95% CI: -0.047, 0.016, p = 0.3279). In the relationship between ln(SIRI) and trunk BMD, when ln(SIRI) < -0.146, each unit increase in ln(SIRI) was associated with a 0.040 g/cm² increase in trunk BMD (β: 0.040; 95% CI: 0.020, 0.060, p < 0.0001). However, when ln(SIRI) > -0.146, the increase in ln(SIRI) did not significantly affect trunk BMD (β: -0.012; 95% CI: -0.035, 0.010, p = 0.2796). For total BMD, when ln(SIRI) < -0.197, each unit increase in ln(SIRI) was associated with a 0.032 g/cm² increase in total BMD (β: 0.032; 95% CI: 0.013, 0.050, p = 0.0011). When ln(SIRI) > -0.197, the increase in ln(SIRI) did not significantly affect total BMD (β: -0.008; 95% CI: -0.027, 0.012, p = 0.4475). Participants were divided into three groups based on total calcium levels (7.30–9.499 mg/dL, 9.50–9.68 mg/dL, 9.681–11.3 mg/dL), and smooth curve and threshold effect evaluations were conducted (**Table 5**, **Figure 4C**). The analysis revealed that in the 7.30–9.499 mg/dL group, the threshold effects between ln(SIRI) and pelvic, trunk, and total BMD were most pronounced. For total BMD, when ln(SIRI) < 0.255, each unit increase in ln(SIRI) was associated with a 0.020 g/cm² increase in total BMD (β: 0.020; 95% CI: 0.006, 0.035, p = 0.0057). In contrast, when ln(SIRI) > 0.255, the increase in ln(SIRI) was significantly negatively correlated with total BMD, with each unit increase in ln(SIRI) associated with a 0.042 g/cm² decrease in total BMD (β: -0.042; 95% CI: -0.067, -0.017, p = 0.0011). Notably, in this total calcium group, only total BMD exhibited a significant threshold effect, while the threshold effect analysis for pelvic and trunk BMD did not reach statistical significance (p > 0.05). This suggests that at lower total calcium levels, increases in ln(SIRI) have a more pronounced effect on total BMD, particularly showing a significant negative effect above the threshold.

## Discussion

This study, using data from the NHANES 2011-2016, explored the association between the Systemic Inflammation Response Index (SIRI) and BMD in children and adolescents aged 8–19. To our knowledge, this is the first study to investigate the relationship between SIRI levels and BMD during the crucial bone growth phases of childhood and adolescence, using a large, nationally representative sample in a cross-sectional design. Our findings indicate a significant association between SIRI levels and BMD, showing a nonlinear relationship, threshold effects, and saturation effects. These results provide new insights into the complex interactions between systemic inflammation and bone health, particularly during key stages of skeletal growth. According to research by Teresa Iantomas et al., in postmenopausal women with osteoporosis, elevated peripheral blood monocyte levels spontaneously differentiate into osteoclasts and secrete pro-inflammatory cytokines such as IL-1, IL-6, and TNF-α, which further stimulate osteoclast differentiation and activity, leading to an increase in osteoclast numbers and enhanced bone resorption (16, 17). The increased expression of nuclear factor kappa B (NF-κB) ligand receptor activator (RANKL) and RANK in inflammatory neutrophils is closely associated with decreased BMD and increased osteoclast bone resorption (18). T lymphocytes and B lymphocytes participate in bone metabolism by secreting cytokines and regulating the RANKL/OPG balance (19–21). T cells promote osteoclastogenesis by expressing RANKL and secreting pro-inflammatory cytokines such as IL-17 and TNF-α, while B cells, in an inflammatory environment, secrete GCSF and RANKL to accelerate bone resorption. Regulatory T cells (Tregs) and regulatory B cells (Bregs) secrete anti-inflammatory cytokines such as TGF-β and IL-10 to suppress osteoclast activity and promote osteoblast function, thus maintaining the dynamic balance of bone homeostasis (22, 23). This complex balance mechanism determines the dual role of lymphocytes in regulating bone density.

Chronic low-grade inflammation is a core factor in the development of osteoporosis, and its significant role in the pathophysiological mechanisms primarily lies in its disruption of the dynamic balance of bone metabolism (24). Recent studies have emphasized the key role of inflammatory cytokines and immune cells in regulating bone metabolism, which may help explain the complex relationship between systemic inflammation and BMD. Specifically, studies on RANKL, a key regulator of osteoclastogenesis, have shown that its expression is significantly regulated by inflammatory cytokines such as TNF-α, IL-6, and IL-1. Under systemic inflammatory conditions, elevated levels of these cytokines can promote osteoclast differentiation, leading to increased bone resorption and a decrease in BMD (25, 26). Furthermore, research on the role of bone marrow endothelial cells (BMECs) in bone homeostasis suggests that these cells not only regulate vascularization but also influence osteoblast and osteoclast activity through the secretion of angiocrine factors, which may further complicate the inflammatory pathways affecting bone health (27). Recent studies have shown that inflammatory markers such as neutrophil/lymphocyte ratio (NLR), monocyte/lymphocyte ratio (MLR), platelet/lymphocyte ratio (PLR), systemic immune-inflammatory index (SII), and SIRI have important clinical value in reflecting systemic inflammatory status, particularly in the study of diseases such as osteoporosis, cardiovascular diseases, cancer, and metabolic syndrome (28, 29). These composite inflammatory indices stand out for their simplicity and cost-effectiveness, and can be easily obtained through routine blood tests. Additionally, they have significant advantages in terms of ease of measurement and stable data, making them widely applied in clinical practice and scientific research (30).

Our study shows that participants in the highest quartile of SIRI (Q4) were significantly older, engaged in more days of moderate-to-vigorous physical activity (MVPA) per week, and had lower levels of total calcium, phosphorus, and ALP compared to those in the lowest quartile (Q1). The observation that participants with higher SIRI levels are older aligns with the trend of increasing systemic inflammation with age (31, 32), which holds true even in children and adolescents. Adolescence is a key period of growth and hormonal fluctuations, closely linked to dynamic changes in immune function and metabolic processes. This may explain why older participants tend to have higher SIRI levels. The age-related increase in systemic inflammation could partially explain the relationship between SIRI and bone density, as older adolescents are closer to the formation stage of peak bone mass (the point at which bone mineral density and bone strength reach their highest levels) (33, 34). During this stage, inflammatory mediators may play an important role in bone metabolism by modulating the dynamic balance between osteoblasts and osteoclasts. The relationship between physical activity and SIRI levels may reflect the combined effects of short-term acute inflammatory responses and long-term anti-inflammatory effects. This phenomenon may be due to a combination of factors, such as the temporary physiological stress induced by high-intensity exercise, the long-term anti-inflammatory effects that are masked by short-term responses, and the stimulatory effects of exercise on bone metabolism. Mechanical loading from physical activity may both enhance bone formation (35, 36) and transiently increase the release of inflammatory mediators, though the specific mechanisms require further investigation. The lower levels of phosphorus and ALP in participants with higher SIRI levels suggest that systemic inflammation may exacerbate bone metabolic imbalance by affecting mineral metabolism and osteoblast function (37), highlighting the importance of controlling inflammation to maintain bone health. Although our results indicate a significant association between SIRI and BMD, after adjusting for BMI and body weight, the effect of SIRI on BMD was no longer significant. This finding suggests that BMI and body weight may play a more critical role in predicting BMD, possibly by modulating inflammatory responses or directly affecting bone metabolism. Previous studies have identified BMI and body weight as important predictors of BMD in children and adolescents, which is consistent with our results. Therefore, we believe that the potential impact of BMI and body weight on SIRI in studies of BMD in children and adolescents warrants further investigation.

In subgroup analysis, the relationship between SIRI and BMD differed significantly by gender, with a stronger positive correlation observed in males compared to females. This difference may be partly due to hormonal and metabolic differences between adolescent males and females during puberty (38). For example, testosterone in males not only promotes bone growth but may also enhance osteoblast activity and sensitivity to inflammatory signals, thereby reinforcing the positive effects of bone metabolism (39, 40). In contrast, estrogen in females has anti-inflammatory and bone-protective effects (41), which may modulate the impact of SIRI on bone metabolism, leading to a weaker effect of SIRI on BMD in females. Saturation and threshold effect analyses further revealed gender- and site-specific effects of SIRI on BMD. In males, SIRI exhibited a saturation effect in the pelvis, trunk, and total BMD, with a significant positive correlation with bone density below a specific threshold. However, once the threshold was exceeded, this positive effect plateaued or became nonsignificant, suggesting that moderate inflammation may promote bone formation, but high levels of inflammation may no longer be beneficial. In females, the threshold effect was observed only for trunk BMD, where SIRI had a significant positive effect on BMD below the threshold, but after surpassing the threshold, an increase in SIRI was associated with a decrease in bone density, indicating that high levels of inflammation may disrupt bone metabolic balance. Age stratification analysis further showed that the positive correlation between SIRI and BMD was most significant in the 14-16year age group. This age range corresponds to the critical period of PBM formation. According to research by Bonjour JP (42) and others, bone metabolism is highly active during this period, with the bone formation rate peaking, making this stage more sensitive to systemic inflammation and other external factors. Moreover, BMI stratification revealed that in the obese group (BMI ≥ 25 kg/m²), the negative effect of SIRI on total BMD was significant (P < 0.05), further supporting the significant role of chronic inflammation in individuals with high BMI. This finding emphasizes the interaction between BMI and inflammation in bone health, suggesting that a combined approach of controlling both BMI and inflammation may be more effective in improving bone mineral density in obese children and adolescents. Additionally, in the interaction test between BMI and SIRI, subgroup analyses stratified by age and different genders showed that, in the higher BMI group, SIRI is an independent predictor of total BMD. The independent predictive role of SIRI on bone density may be modulated by BMI and gender, particularly in the obese population (BMI ≥ 25), where its effect is more pronounced. This also supports the idea that obesity, as a progressive disease, may alter the impact of inflammatory status on BMD. In terms of age, in the younger age groups (8-10 years and 11-13 years), the significant effect of SIRI on bone density was concentrated in total BMD, while in the older age groups (14-16 years and 17-19 years), the effects were more complex, showing both significant positive and negative effects. Notably, total BMD was the site most significantly influenced by SIRI, and the combined modulating effects of BMI and age were particularly evident at this site, suggesting that the independent predictive role of SIRI is crucial in specific BMI and age subgroups.

The positive correlation observed between SIRI and BMD in this study is significant because it contrasts sharply with the negative correlation commonly reported in adult populations. In adults, chronic systemic inflammation is typically associated with increased bone resorption and decreased bone formation (43–45), leading to a reduction in bone density. However, this study shows a positive correlation between SIRI levels and pelvic, trunk, and total BMD, independent of lumbar spine BMD. This may be due to differences in metabolic characteristics of different skeletal regions and their sensitivity to systemic inflammatory signals. After adjusting for covariates, this positive correlation remained stable, suggesting that elevated SIRI levels during childhood and adolescence may contribute to an increase in BMD. Notably, this positive correlation is not infinite. Analysis revealed that beyond a certain threshold (e.g., -0.336 for pelvic BMD), the relationship gradually plateaued and even reversed in some cases, especially for total BMD. This saturation effect suggests that systemic inflammation may have a dual role: during the skeletal growth phase, moderate inflammation levels could promote bone remodeling by modulating the activity of bone metabolism-related cells (such as osteoclasts and osteoblasts). However, when inflammation levels exceed a certain threshold, prolonged high systemic inflammation may disrupt the dynamic balance of bone remodeling, inhibiting bone formation or accelerating bone resorption. This phenomenon is consistent with the hypothesis that low-level systemic inflammation in children and adolescents may stimulate bone metabolism through inflammatory cytokines (e.g., TNF-α, IL-6), which regulate the activity of osteoclasts and osteoblasts. This finding provides new insights into the mechanisms by which inflammation and bone metabolism interact at different ages and underscores the importance of maintaining moderate inflammation levels for bone health in children and adolescents. Furthermore, the relationship between SIRI levels and total BMD is significantly modulated by total calcium levels, with individuals in the lowest calcium quartile showing a pronounced threshold effect. At low calcium levels, moderate systemic inflammation may exert a protective effect by promoting bone metabolism, but once inflammation exceeds a threshold, the balance of bone metabolism is disrupted, leading to a significant decrease in bone density. In contrast, individuals with higher calcium levels did not exhibit a significant threshold effect, suggesting that adequate calcium intake may stabilize bone metabolism dynamics, effectively buffering the negative impact of high inflammation levels on BMD. These results indicate that populations with low calcium intake are more sensitive to the negative effects of systemic inflammation, emphasizing the importance of increasing calcium intake and managing inflammation for bone health protection, especially for high-risk groups with inadequate calcium intake (35, 46, 47).

This study has several limitations. First, due to its cross-sectional design, we cannot establish a causal relationship between SIRI levels and BMD in adolescents. Second, data limitations prevented the inclusion of all possible covariates that might influence bone metabolism, so potential confounding factors, such as dietary habits, types of physical activity, and genetic background, may still exist. Third, although the study population is based on the NHANES national sample, which is representative, the external validity of the results may be limited, especially in groups with different demographic characteristics or healthcare systems. Additionally, NHANES data did not include some key inflammatory biomarkers, which limits a comprehensive analysis of the inflammatory mechanisms. Therefore, future longitudinal studies and multi-center cohort studies will help further explore the causal relationship between SIRI and bone density and provide more insight into the underlying biological mechanisms.

## Conclusion

This study reveals the relationship between SIRI and BMD in children and adolescents aged 8 to 19 years. The analysis shows a significant positive correlation between SIRI levels and BMD in the pelvis, trunk, and total body, with this relationship demonstrating nonlinearity and saturation effects. Furthermore, subgroup analysis suggests that factors such as sex, age, and BMI may play a moderating role in the relationship between SIRI and BMD. Overall, SIRI is closely related to BMD, with its effects varying across different age groups, sexes, and BMI categories, providing new insights into the inflammatory mechanisms underlying bone density development in children and adolescents. In conclusion, this study suggests that SIRI could be a valuable biomarker, not only as an early tool for predicting changes in bone density but also for identifying high-risk individuals, thus guiding personalized interventions and optimizing bone health management strategies. This finding offers a simple, cost-effective, and widely applicable method for clinical use, potentially playing a key role in the prevention and treatment of bone diseases such as osteoporosis in the future.

## Data Availability

The original contributions presented in the study are included in the article/**Supplementary Material**. Further inquiries can be directed to the corresponding author.

## Supplementary material

Supplementary Table 1 The Relationship Between Ln SIRI and BMD After Adjusting for BMI as a Covariate.

Supplementary Table 2 The Relationship Between Ln SIRI and BMD After Adjusting for weight as a Covariate.
