# Supplementary material for: Relationship between systemic inflammatory response index and bone mineral density in children and adolescents aged 8-19 years: a cross-sectional study based on NHANES 2011-2016
Source: Front Endocrinol (Lausanne). 2025 Feb 25;16:1537574. doi: 10.3389/fendo.2025.1537574 (PMC11893988; doi:10.3389/fendo.2025.1537574)
Supplement: Supplementary Table 2 — The Relationship Between Ln SIRI and BMD After Adjusting for weight as a Covariate. [file Table2.docx]

Supplementary Table 2. The Relationship Between Ln SIRI and BMD After Adjusting for weight as a Covariate.

| Exposure | Model 1[β (95%CI)] | Model 2[β (95%CI)] | Model 3[β (95%CI)] |
| --- | --- | --- | --- |
| Lumbar BMD (continuous) | 0.038 (0.028, 0.048) | 0.011 (0.004, 0.017) | -0.004 (-0.010, 0.002) |
| Lumbar BMD (quartile) | | | |
| Quartile 1 | Reference | Reference | Reference |
| Quartile 2 | 0.020 (0.002, 0.039) | 0.015 (0.003, 0.027) | 0.005 (-0.006, 0.016) |
| Quartile 3 | 0.054 (0.036, 0.073) | 0.025 (0.013, 0.036) | 0.007 (-0.004, 0.018) |
| Quartile 4 | 0.080 (0.061, 0.098) | 0.028 (0.016, 0.040) | 0.003 (-0.008, 0.014) |
| p for trend | <0.00001 | 0.00123 | 0.16176 |
| Pelvis BMD (continuous) | 0.056 (0.044, 0.069) | 0.025 (0.016, 0.033) | -0.003 (-0.010, 0.004) |
| Pelvis BMD (quartile) | | | |
| Quartile 1 | Reference | Reference | Reference |
| Quartile 2 | 0.041 (0.018, 0.064) | 0.035 (0.019, 0.050) | 0.015 (0.002, 0.027) |
| Quartile 3 | 0.073 (0.050, 0.095) | 0.039 (0.024, 0.055) | 0.008 (-0.005, 0.020) |
| Quartile 4 | 0.109 (0.086, 0.132) | 0.050 (0.034, 0.065) | 0.003 (-0.010, 0.016) |
| p for trend | <0.00001 | <0.00001 | 0.41854 |
| Trunk BMD (continuous) | 0.032 (0.024, 0.040) | 0.011 (0.005, 0.016) | -0.004 (-0.009, 0.001) |
| Trunk BMD (quartile) | | | |
| Quartile 1 | Reference | Reference | Reference |
| Quartile 2 | 0.020 (0.005, 0.036) | 0.016 (0.006, 0.026) | 0.006 (-0.003, 0.014) |
| Quartile 3 | 0.042 (0.027, 0.057) | 0.020 (0.010, 0.030) | 0.004 (-0.005, 0.012) |
| Quartile 4 | 0.063 (0.048, 0.079) | 0.023 (0.013, 0.033) | -0.001 (-0.010, 0.008) |
| p for trend | <0.00001 | 0.00014 | 0.13190 |
| Total BMD (continuous) | 0.031 (0.023, 0.039) | 0.010 (0.005, 0.015) | -0.004 (-0.008, 0.001) |
| Total BMD (quartile) | | | |
| Quartile 1 | Reference | Reference | Reference |
| Quartile 2 | 0.020 (0.005, 0.035) | 0.017 (0.007, 0.026) | 0.007 (-0.001, 0.015) |
| Quartile 3 | 0.040 (0.025, 0.055) | 0.020 (0.011, 0.029) | 0.005 (-0.003, 0.013) |
| Quartile 4 | 0.061 (0.046, 0.076) | 0.021 (0.012, 0.031) | -0.001 (-0.010, 0.007) |
| p for trend | <0.00001 | 0.00010 | 0.12124 |
